# Block Versus Particulate Deproteinized Bovine Bone Mineral for Guided Bone Regeneration of Peri‐Implant Dehiscence Defects: A 5‐Year Randomized Controlled Trial

**DOI:** 10.1111/clr.70150

**Published:** 2026-06-28

**Authors:** Anina N. Zuercher, Erkan Evci, Riccardo D. Kraus, Ronald E. Jung, Franz J. Strauss, Goran Benic

**Affiliations:** ^1^ Clinic of Reconstructive Dentistry, Center of Dental Medicine University of Zurich Zurich Switzerland; ^2^ Universidad Autonoma de Chile Santiago Chile; ^3^ Faculty of Dentistry, Universidad Finis Terrae Santiago Chile

**Keywords:** buccal hard‐tissue thickness, deproteinized bovine bone mineral blocks, guided bone regeneration, peri‐implant dehiscence defects

## Abstract

To assess the 5‐year dimensional stability of buccal hard‐tissues following guided bone regeneration (GBR) of peri‐implant dehiscence defects using deproteinized bovine bone mineral (DBBM) blocks or particulate.

**Methods:**

Patients requiring single‐tooth implants and presenting a buccal dehiscence defect (≥ 3 mm) at implant placement were randomly allocated to GBR using either particulate DBBM or an individually shaped DBBM‐block, both covered with a resorbable collagen membrane. Buccal hard‐tissue thickness was assessed using cone‐beam computed tomography postoperatively, at 6 months and at 5 years. Buccal horizontal thickness at the implant shoulder and corono‐buccal thickness measured at 45° were recorded. Secondary outcomes included marginal bone levels, clinical parameters and implant survival. Linear mixed‐effects models were used to compare the groups to account for within‐subject correlations.

**Results:**

Twenty‐two of 24 patients completed the 5‐year follow‐up. Buccal hard‐tissue thickness decreased over time in both groups, however, the block‐group showed significantly greater dimensional stability. At 5 years, mean buccal horizontal hard‐tissue thickness was 1.36 ± 1.16 mm in the block‐group and 0.07 ± 0.16 mm in the particulate‐group (adjusted mean difference, −1.2 mm; 95% CI, −1.9 to−0.6; *p* < 0.001). Corono‐buccal thickness at 45° was also significantly greater in the block‐group (adjusted mean difference, −0.3 mm; 95% CI, −0.5 to −0.1; *p* = 0.002). Marginal bone levels and clinical parameters showed no clinically relevant differences. No implant loss or peri‐implantitis was observed.

**Conclusion:**

GBR using either DBBM blocks or particulate DBBM resulted in similar peri‐implant clinical outcomes and implant survival at 5 years. However, DBBM blocks provided superior long‐term preservation of the buccal hard‐tissue contour.

**Trial Registration:**

Clinical Trial Registration Number, DRKS00005803

## 1 Introduction

Guided bone regeneration (GBR) is a well‐established procedure to augment peri‐implant defects and for enabling implant placement in a prosthetically driven position (Benic and Hammerle 2014) (Dahlin et al. 1988). The biological rationale of GBR relies on the use of a barrier membrane to exclude soft‐tissue cell ingrowth while maintaining a protected space that allows repopulation by osteogenic cells and subsequent hard‐tissue regeneration. In esthetically demanding areas, GBR is frequently performed to re‐establish or enhance the buccal contour of the alveolar ridge, thereby improving long‐term functional and esthetic outcomes (De Bruyckere et al. 2020).

Currently, the most common GBR approach combines a resorbable collagen membrane with a particulate bone substitute (Omar et al. 2019). Although this combination is widely used and clinically successful, systematic reviews indicate substantial variability in defect resolution and long‐term volume stability. Mean vertical defect resolution of around 81% has been described, with reported ranges between 56% and 97% (Thoma et al. 2019). This wide variability highlights the sensitivity of GBR outcomes to multiple factors including defect morphology and, most critically, the ability to maintain space during flap closure and early healing.

A key limitation of particulate grafts is their susceptibility to displacement when exposed to flap pressure and soft‐tissue tension with flap closure, during wound closure, potentially compromising the augmented volume. Preclinical and in vitro studies have shown that flap closure alone can induce significant collapse of the grafted area when particulate materials are used, even in the presence of membrane coverage and fixation devices (Mellonig et al. 1998; Mir‐Mari et al. 2017, 2016). These observations have driven continued interest in biomaterials that provide greater mechanical resistance and improved space maintenance.

In this context, bone substitute blocks, particularly deproteinized bovine bone mineral (DBBM) blocks, have been proposed as a means to better resist compressive forces and preserve the intended augmented contour. Experimental studies have demonstrated that block grafts exhibit reduced displacement during wound closure and improved maintenance of ridge dimensions during early healing compared with particulate materials (Benic et al. 2016; Schwarz et al. 2008) (Benic et al. 2017). These findings were confirmed by a randomized controlled trial (RCT), demonstrating greater buccal horizontal hard‐tissue thickness at 6 months when DBBM blocks were used instead of particulate DBBM for GBR of peri‐implant dehiscence‐type defects (Benic et al. 2019). While early healing outcomes are clinically relevant, long‐term dimensional stability remains the decisive determinant of success, especially in esthetically sensitive implant sites. However, to date, long‐term clinical data are lacking regarding whether the initial advantages of DBBM block grafts are maintained beyond the initial healing period, or whether particulate grafts continue to undergo collapse or eventually stabilize over time.

Therefore, the aim of the present study was to assess the 5‐year dimensional stability of hard tissues augmented with either DBBM block or particulate DBBM in the context of GBR for peri‐implant dehiscence defects.

## 2 Materials and Methods

### 2.1 Study Design

The present clinical trial represents the 5‐year follow‐up of a previously published RCT with two parallel treatment arms (Benic et al. 2019). This study was performed at the Clinic of Reconstructive Dentistry, Center of Dental Medicine, University of Zurich, Switzerland. Patients in need of a dental implant and presenting a buccal dehiscence defect at the time of surgery were randomly allocated to one of two GBR treatment modalities (Block versus Granules). The study protocol was approved by the Cantonal Ethics Committee of Zurich (reference code KEK‐ZH2011‐0075) and registered in the German Clinical Trials Register (DRKS00005803). All procedures performed in this study involving human participants were conducted in accordance with the Declaration of Helsinki. All participants provided written informed consent prior to enrolment.

### 2.2 Study Population

A detailed description of the study design and recruitment process has been published previously (Benic et al. 2019). In brief, the original randomized controlled clinical trial included adult patients (≥ 18 years) in need of a single‐tooth implant in the maxilla or mandible who presented a buccal dehiscence defect at the time of implant placement. Patients were eligible if they were in good general and periodontal health, demonstrated full‐mouth plaque and bleeding scores below 25%, and showed probing depths < 4 mm. Additional requirements included the presence of at least one adjacent tooth and an opposing dentition. Patients were excluded if they presented systemic conditions affecting bone or soft‐tissue healing, were pregnant or breastfeeding, had active periodontal disease, smoked more than 15 cigarettes per day, or exhibited poor oral hygiene or insufficient compliance. For the present study, only patients from the original RCT who completed the 5‐year follow‐up examination and had complete CBCT datasets were included in the final analysis.

### 2.3 Clinical Procedures

The surgical procedures have been described in detail in the original publication (Benic et al. 2019). Briefly, implant placement was performed under local anesthesia following a standardized surgical protocol. After an intrasulcular and vertical releasing incision, a full‐thickness mucoperiosteal flap was elevated to expose the alveolar crest and the peri‐implant defect morphology. Implant osteotomies were prepared according to the manufacturer's recommendations, and a titanium implant (AstraTech/OsseoSpeed EV, Dentsply Sirona Implants) was placed in a prosthetically ideal position.

**FIGURE 1:**
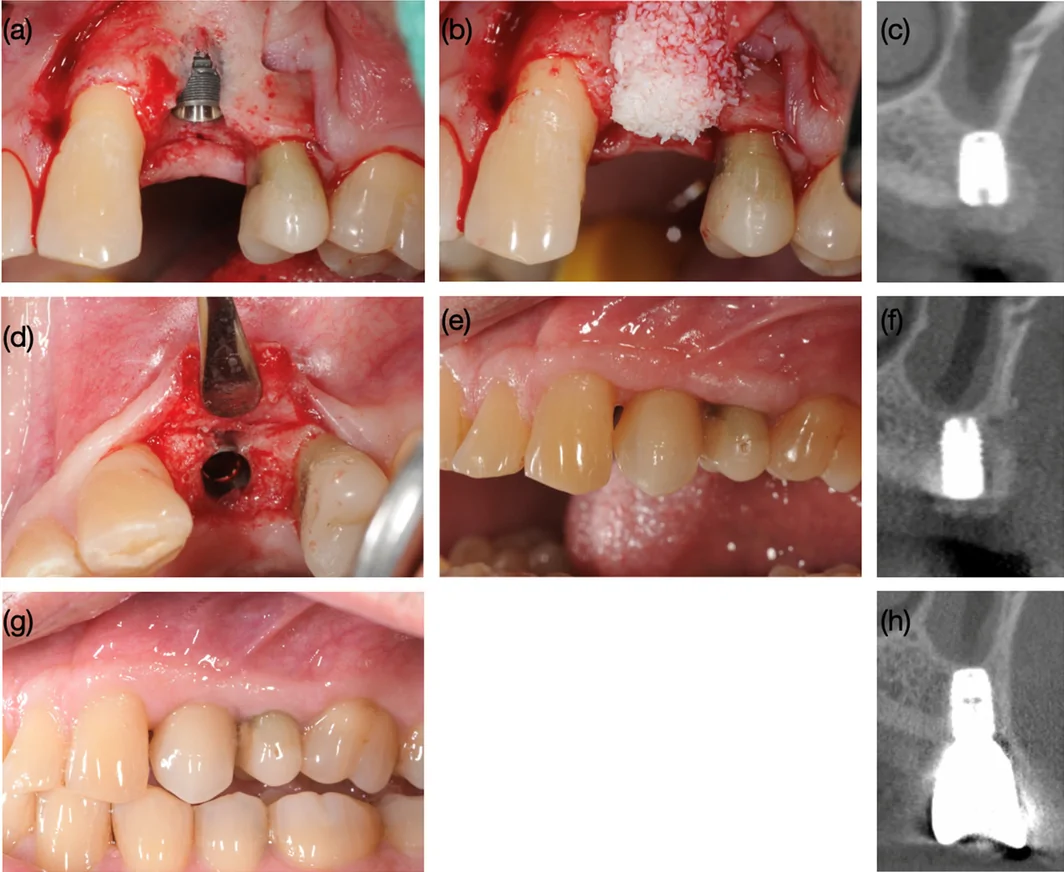
Representative case of a peri‐implant dehiscence defect treated with particulate DBBM (a) Clinical buccal view of the dehiscence defect immediately after implant placement. (b) Same site after defect filling with particulate deproteinized bovine bone mineral, prior to coverage with a resorbable collagen membrane stabilized with polylactide fixation pins. (c) Bucco‐oral CBCT cross‐section immediately after wound closure (post‐operative baseline). (d) Intra‐operative buccal view at re‐entry surgery, 6 months after implant placement, showing incomplete vertical defect resolution. (e) Clinical buccal view of the implant‐supported restoration at the 1‐year follow‐up. (f) Bucco‐oral CBCT cross‐section at the 6‐month follow‐up. (g) Clinical buccal view of the implant‐supported restoration at the 5‐year follow‐up. (h) Bucco‐oral CBCT cross‐section at the 5‐year follow‐up.

**FIGURE 2:**
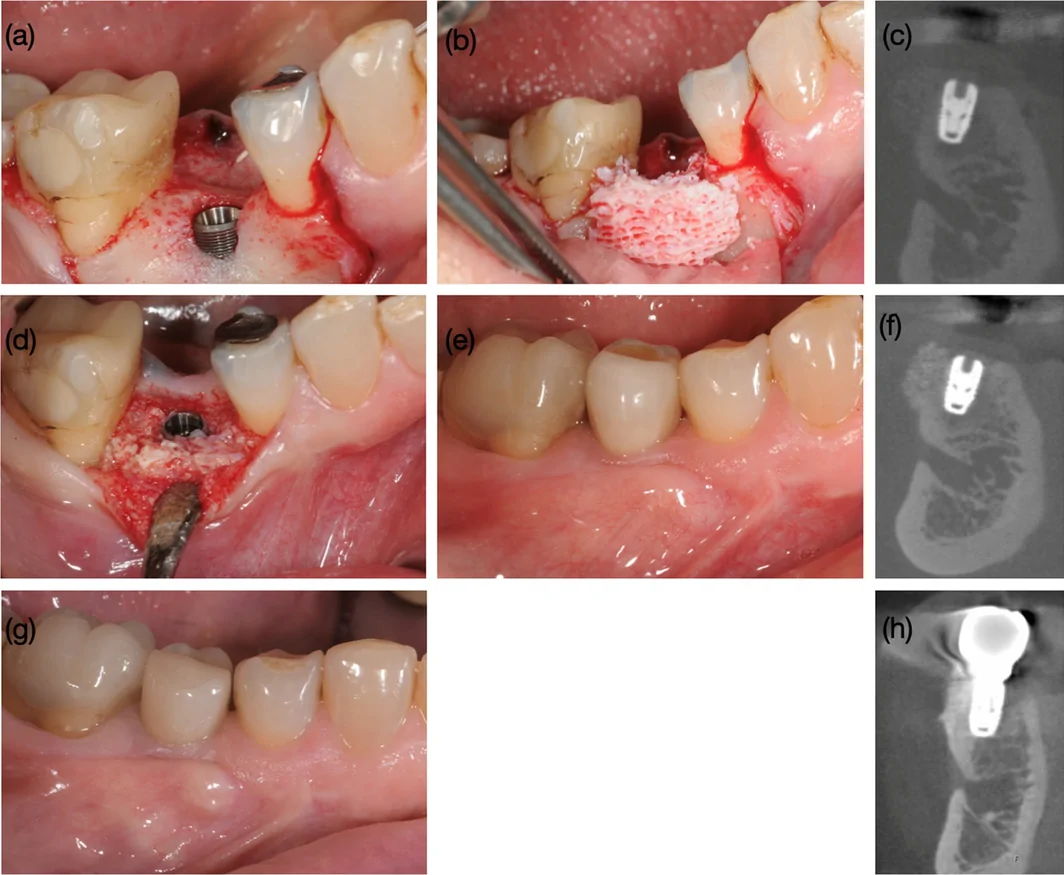
Representative case of a peri‐implant dehiscence defect treated with a DBBM block (a) Clinical buccal view of the dehiscence defect immediately after implant placement. (b) Same site after defect filling with an individually shaped deproteinized bovine bone mineral block, prior to coverage with a resorbable collagen membrane stabilized with polylactide fixation pins. (c) Bucco‐oral CBCT cross‐section immediately after wound closure (post‐operative baseline). (d) Intra‐operative buccal view at re‐entry surgery, 6 months after implant placement, showing complete vertical defect resolution. (e) Clinical buccal view of the implant‐supported restoration at the 1‐year follow‐up. (f) Bucco‐oral CBCT cross‐section at the 6‐month follow‐up. (g) Clinical buccal view of the implant‐supported restoration at the 5‐year follow‐up. (h) Bucco‐oral CBCT cross‐section at the 5‐year follow‐up.

Following implant placement, the buccal dehiscence defect was measured using a calibrated periodontal probe. Only defects with a vertical height ≥ 3 mm were eligible for inclusion (Figures 1 and 2). Sites were then randomly allocated to one of two GBR modalities:

1. GBR using particulate DBBM, or
2. GBR using an individually shaped DBBM block.

In both groups, augmentation followed a standardized protocol. Defects were filled with either DBBM granules (Bio‐Oss spongiosa, 0.25–1 mm, Geistlich Pharma AG, Wolhusen, Switzerland) or an extraorally shaped DBBM block (Bio‐Oss block, Geistlich Pharma AG, Wolhusen, Switzerland). A native bilayer collagen membrane (Bio‐Gide Geistlich Pharma AG, Wolhusen, Switzerland) was adapted to overlap the defect margins by at least 2 mm and secured with resorbable polylactide fixation pins (Inion; Inion Oy, Tampere, Finland).

At 6 months, a re‐entry procedure was performed (Figures 1 and 2). Cover screws were replaced by healing abutments and the flaps were sutured around the abutment. Sutures were removed after 1 week. Subsequently, implant‐level impressions were taken and a screw‐retained monolithic zirconia crown on a titanium base was delivered. In esthetically demanding sites, buccal veneering of the monolithic zirconia crown with feldspathic porcelain was performed when indicated. Screw‐access channel was sealed with Teflon tape and resin composite. All patients were enrolled in a structured maintenance program, consisting of professional prophylaxis and regular clinical monitoring throughout the entire follow‐up period.

### 2.4 Baseline and Clinical Follow‐Up Examinations

Clinical assessments were performed pre‐ and post‐operatively, including at the time of re‐entry surgery (6 months after implant placement). Follow‐up examinations were conducted at 1 year and 5 years after implant placement (Figures 1 and 2).

### 2.5 Outcome Measures

#### 2.5.1 Primary Outcome: Bucco‐Oral Hard Tissue Thickness

Cone‐beam computed tomography (CBCT) scans were obtained immediately after implant placement, at 6 months and at the 5‐year follow‐up. Imaging was performed with a 3D Accuitomo 170 scanner (J. Morita Europe GmbH) using standardized settings (90 kV, 5 mA, voxel size 0.125–0.25 mm). A bucco‐oral cross‐sectional slice perpendicular to the implant axis was generated for analysis using software (i‐Dixel 3D, J. Morita Europe GmbH). Standardized linear measurements were performed on each scan to quantify the dimension of the augmented buccal hard tissue:

- **Horizontal thickness (HT):** linear distance from the outer contour of the buccal hard tissue to the implant shoulder, measured perpendicular to the long axis of the implant.
- **45‐degree thickness (45°T):** linear distance from the implant shoulder to the buccal bone surface measured along a line oriented 45° coronally to the implant axis, reflecting corono‐buccal contour of the regenerated hard tissue

**FIGURE 3:**
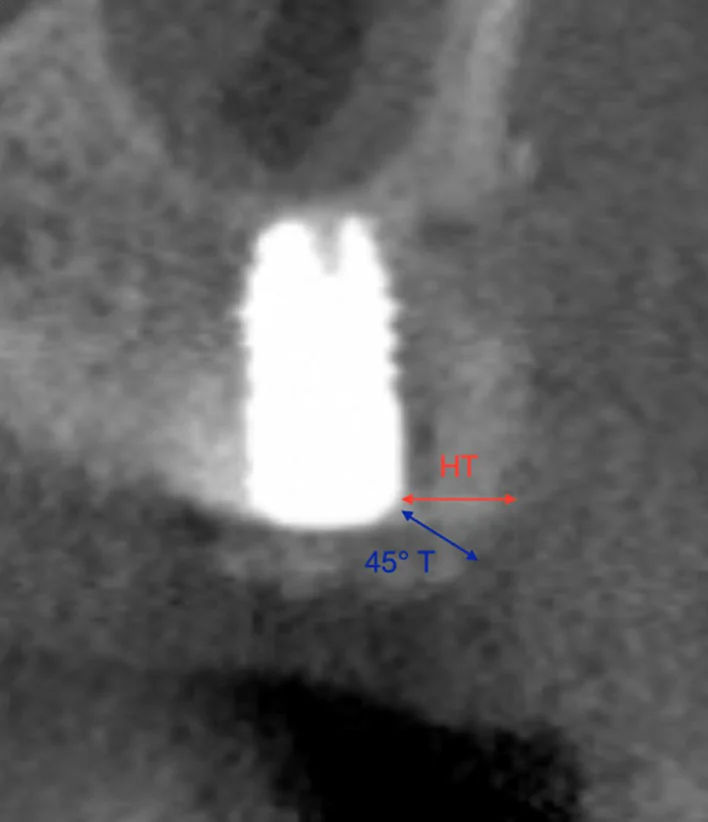
Schematic illustration of the two radiographic linear measurements assessed on the bucco‐oral CBCT cross‐section. The horizontal thickness (HT) corresponds to the distance from the implant shoulder to the outer contour of the buccal hard tissue, perpendicular to the implant axis. The 45° thickness (45°T) corresponds to the distance from the implant shoulder to the buccal hard‐tissue surface, along a line oriented 45° coronally to the implant long axis.

All linear measurements were performed on the cross‐sectional CBCT slice located at the mid‐buccal position of the implant, that is, the section perpendicular to the long axis of the implant and closest to the buccal bone wall. Both measurements quantify the buccal hard tissue around the implant on CBCT. The 45° corono‐buccal measurement was specifically selected because the hard tissue in the coronal‐buccal region—coronal to the implant shoulder—is the structure that supports the buccal volume and, consequently, the overlying soft‐tissue contour and the prosthetic emergence profile, while the horizontal measurement at the implant shoulder captures the hard‐tissue dimension at a more apical level. A schematic illustration of both measurements is provided in Figure 3.

#### 2.5.2 Marginal Bone Level

Marginal bone levels (MBL) were evaluated at 6 months and 5 years follow‐up. Standardized periapical radiographs were obtained using a paralleling technique. The distance from the implant shoulder to the first bone‐to‐implant contact (fBIC) was measured on the mesial and distal aspects. The known thread pitch of the implant was used for calibration of all apico‐coronal measurements. Radiographs were analyzed using open‐source image analysis software (ImageJ, National Institutes of Health, Bethesda, MD, USA).

#### 2.5.3 Clinical and Periodontal Parameters

At the 1‐year and 5‐year follow‐up visits, clinical and periodontal parameters were recorded at six sites per implant (mesio‐buccal, mid‐buccal, disto‐buccal, mesio‐oral, mid‐oral, and disto‐oral) using a calibrated periodontal probe (PCPUNC 15, Hu‐Friedy, Chicago, IL, USA). The following parameters were assessed: probing depth (PD), bleeding on probing (BOP), recorded as present or absent within 15 s after gentle probing; plaque index (PI), documented dichotomously as the presence or absence of visible plaque; and the width of keratinized mucosa (KM), measured mid‐facially. Implant survival was defined as the absence of mobility, persistent pain, or radiographic radiolucency. Implant survival was defined as the implant being functionally in situ at the 5‐year follow‐up examination.

##### 2.5.3.1 Diagnosis of Peri‐Implant Conditions

Peri‐implantitis was diagnosed according to the case definitions of the 2017 World Workshop on the Classification of Periodontal and Peri‐implant Diseases and Conditions (Berglundh et al. 2018). Diagnosis required the simultaneous presence of: (i) bleeding and/or suppuration on gentle probing (probing force ≤ 0.25 N), (ii) increased probing depth compared to baseline (6‐month) measurements, and (iii) progressive radiographic bone loss compared with the 6‐month reference radiograph, exceeding crestal bone‐level changes attributable to initial bone remodelling. In the absence of complete baseline reference data, peri‐implantitis was diagnosed when bleeding/suppuration on gentle probing was present in combination with probing depths ≥ 6 mm and radiographic bone levels ≥ 3 mm apical of the most coronal portion of the intraosseous part of the implant. Peri‐implant mucositis was defined according to the updated ID‐COSM consensus (Tonetti et al. 2023) (Herrera et al. 2023) as bleeding on probing at more than one site on gentle probing in the absence of progressive radiographic bone loss beyond initial crestal bone remodelling. Peri‐implant health was defined as the absence of bleeding/suppuration on probing without signs of bone loss beyond physiological remodelling.

##### 2.5.3.2 Sample Size

The sample size calculation was based on two independent groups assuming a normal distribution and using a two‐sample *t‐*test. A total of 17 patients per group (34 patients overall) was required to detect a difference of 0.5 mm, assuming a standard deviation of 0.5 mm, with 80% power and a significance level of 0.05. Since no previous clinical data were available for this specific combination of materials, the sample size calculation was considered exploratory.

##### 2.5.3.3 Statistical Analysis

Descriptive statistics were computed for all parameters means, standard deviations (SD) and medians were reported for continuous variables, while frequencies were used for categorical variables. Changes in clinical and radiographic outcomes, both within and between treatment groups, were analyzed using linear mixed‐effects models, which accounted for within‐subject correlations due to repeated measurements. Fixed effects included treatment group, time and their interaction, enabling the estimation of treatment effects at each time point. Patients were treated as random effects. Group differences were assessed using the linear contrast command. Model assumptions were evaluated visually using residual diagnostics, including Q‐Q plots and histograms. When assumptions were not met, non‐parametric general linear models with generalized estimating equations (GEEs) were applied. A two‐sided significance level of 5% (α = 0.05) was applied throughout. All statistical analyses were performed using Stata v19.5 (StataCorp, College Station, TX).

## 3 Results

### 3.1 Study Population

**FIGURE 4:**
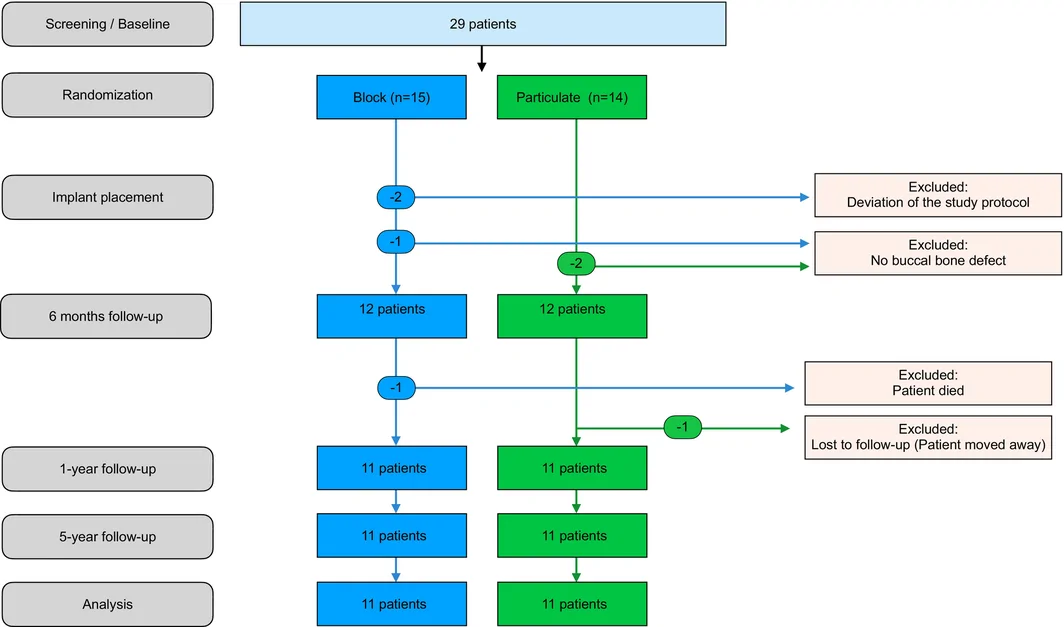
Flow chart diagram.

The CONSORT flow diagram is presented in Figure 4. Of the 24 patients who were analyzed at the 6‐month visit, 22 completed the 5‐year follow‐up. One patient in the particulate group could not be reached and one patient in the block group died during the follow‐up period. All remaining patients were included in the final 5‐year analysis. A detailed summary of patient demographics, implant positions, implant diameters and lengths, and baseline defect dimensions (vertical dehiscence height and horizontal defect width) per treatment group is provided in Table S1.

### 3.2 Buccal Bone Thickness

**FIGURE 5:**
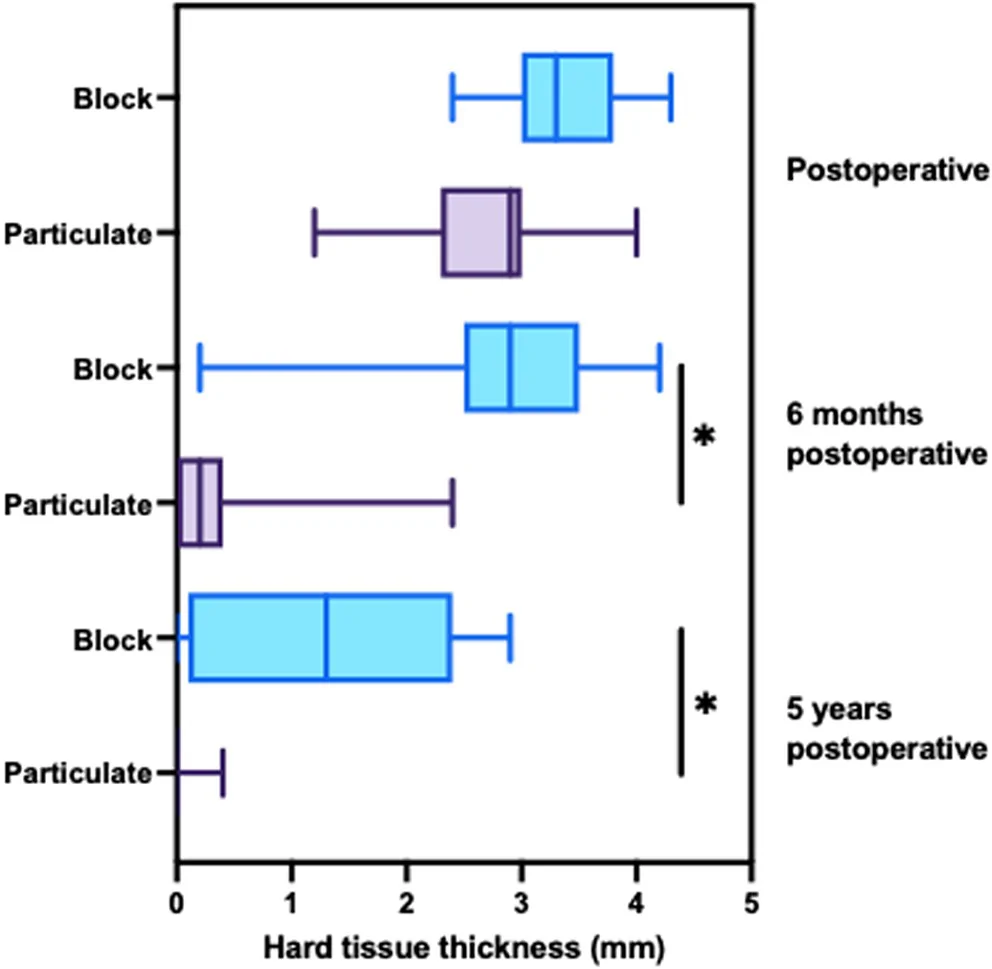
Boxplots representing the buccal horizontal thickness of the augmented hard tissue at different time points. Differences between groups were analyzed using a linear mixed‐effects model. * indicates *p*‐values < 0.05.

Buccal hard‐tissue thickness values at all time points are presented in Table 1 and Table S1 and illustrated in Figure 5. From postoperative to the 5‐year follow‐up, horizontal bone thickness at the implant shoulder decreased in both groups, with significantly greater dimensional stability in the block group (Figure 5). At 5 years, mean horizontal thickness was 1.36 ± 1.16 mm (median 1.30 mm) in the block group and 0.07 ± 0.16 mm (median 0.00 mm) in the particulate group (adjusted mean difference, −1.2 mm 95% CI: −1.9; −0.6; *p* < 0.001) indicating significantly greater buccal hard tissue in the block group (Table 1). Longitudinal analysis demonstrated a significantly smaller horizontal reduction in the block group (−2.01 mm) compared with the particulate group (−2.65 mm), corresponding to the estimated adjusted treatment effect reported above. Negative values indicate a reduction of the buccal bone thickness relative to baseline.

**TABLE 1:** Horizontal and 45° buccal hard‐tissue thickness over time by treatment group and between‐group comparisons.

Parameter	Timepoint	Group block		Group particulate		Comparison	
		Mean (SD)	Median (Q1; Q3)	Mean (SD)	Median (Q1; Q3)	Adjusted mean treatment difference (95% CI)	*p*
**Horizontal thickness**	Post‐OP	3.37 (0.61)	3.30 (3.00, 3.80)	2.72 (0.72)	2.90 (2.30, 3.00)	−0.6 (−1.3; 0.3)	0.063
	6 months	2.69 (1.25)	2.90 (2.50, 3.50)	0.39 (0.70)	0.20 (0.00, 0.40)	−2.3 (−2.9; −1.6)	**< 0.001**
	5 years	1.36 (1.16)	1.30 (0.10, 2.40)	0.07 (0.16)	0.00 (0.00, 0.00)	−1.2 (−1.9; −0.6)	**< 0.001**
**45° thickness**	Post‐OP	2.40 (0.57)	2.20 (3.00, 1.90)	1.91 (0.70)	2.10 (1.70, 2.10)	−0.4 (−1.0; 0.0)	0.067
	6 months	1.53 (0.93)	1.70 (1.70, 2.10)	0.21 (0.53)	0.00 (0.00, 0.20)	−1.3 (−1.9; −0.6)	**< 0.001**
	5 years	0.34 (0.38)	0.10 (0.00, 0.70)	0.00 (0.00)	0.00 (0.00, 0.00)	−0.3 (−0.5; −0.1)	**0.002**

*Note:* Differences were assessed using linear mixed‐effects or GEE models including treatment, time, and their interaction. *p‐*values are reported. Bold values indicate statistically significant differences.

Abbreviations: BOP, bleeding on probing; CI, confidence interval; KM, keratinized mucosa; PD, probing depth; PI, plaque index; PD, probing depth; SD, standard deviation; Q1, First quartile, Q3, Third quartile.

When changes beyond early healing were examined, horizontal bone thickness decreased from 6 months to 5 years, by −1.33 mm in the block group and −0.32 mm in the particulate group. This pattern indicates that most dimensional loss in the particulate group occurred during early healing, whereas block group exhibited a more gradual but continued reduction over time (Figures 1 and 2 and Figure A2).

A similar trend was observed for the 45° corono‐buccal measurement. Thickness values decreased over time in both groups (Table 1). At 5 years, the mean 45° thickness was 0.34 ± 0.38 mm (median 0.10 mm) in the block group and 0.00 ± 0.00 mm (median 0.00 mm) in the particulate group (adjusted mean difference, −0.3 mm 95% CI: −0.5; −0.1; *p* = 0.002), again favoring the block group (Table 1).

### 3.3 Marginal Bone Loss

Marginal bone levels at 6 months and 5 years were comparable between the block and particulate groups (Table 2). At 6 months, both groups showed identical mean MBL values (0.01 ± 0.03 mm), with medians of 0.00 mm. At 5 years, mean MBL measured 0.29 ± 0.33 mm in the block group and 0.07 ± 0.13 mm in the particulate group, corresponding to an adjusted mean difference of −0.22 mm (95% CI −0.43 to 0.00; *p* = 0.051) with no significant differences.

**TABLE 2:** Marginal bone levels and clinical parameters by treatment group and between‐group comparisons.

Parameter	Timepoint	Group block		Group particulate		Comparison	
		Mean (SD)	Median (Q1; Q3)	Mean (SD)	Median (Q1; Q3)	Adjusted mean treatment difference (95% CI)	*p*
**Marginal bone level**	6 months	0.01 (0.03)	0.00 (0.00, 0.00)	0.01 (0.03)	0.00 (0.00, 0.00)	−0.0 (−0.0; 0.0)	0.970
	5 years	0.29 (0.33)	0.13 (0.00, 0.64)	0.07 (0.13)	0.00 (0.00, 0.12)	−0.2 (−0.4; 0.0)	0.051
**BOP**	1 year	0.22 (0.21)	0.17 (0.00, 0.50)	0.22 (0.17)	0.17 (0.17, 0.33)	0.0 (−0.1; 0.1)	0.997
	5 years	0.21 (0.14)	0.17 (0.17, 0.33)	0.19 (0.19)	0.17 (0.00, 0.33)	−0.0 (−0.1; 0.1)	0.840
**Pl**	1 year	0.16 (0.25)	0.00 (0.00, 0.50)	0.06 (0.11)	0.00 (0.00, 0.17)	−0.1 (−0.2; 0.0)	0.201
	5 years	0.06 (0.20)	0.00 (0.00, 0.00)	0.06 (0.15)	0.00 (0.00, 0.00)	0.0 (−0.1; 0.1)	1.000
**PD**	1 year	2.97 (0.89)	3.00 (2.33, 3.17)	2.86 (0.36)	3.00 (2.67, 3.17)	−0.1 (−0.6; 0.3)	0.676
	5 years	3.25 (0.47)	3.30 (2.83, 3.67)	2.68 (0.63)	2.67 (2.17, 3.17)	−0.5 (−1.0; −0.0)	**0.024**
**KM**	1 year	2.18 (0.95)	2.00 (1.00, 3.00)	2.90 (0.83)	2.50 (2.00, 4.00)	0.7 (0.0; 1.4)	0.050
	5 years	1.81 (0.95)	1.50 (1.00, 3.00)	2.27 (0.87)	2.00 (2.00, 3.00)	0.4 (−0.3; 1.3)	0.267

*Note:* Differences were assessed using linear mixed‐effects or GEE models including treatment, time, and their interaction. *p‐*values are reported. Bold values indicate statistically significant differences.

Abbreviations: BOP, bleeding on probing; CI, confidence interval; KM, keratinized mucosa; PD, probing depth; PI, plaque index; SD, standard deviation; Q1, first quartile; Q3, third quartile.

### 3.4 Clinical and Periodontal Parameters

Probing depth (PD) did not show a significant overall group‐by‐time interaction (*p* = 0.16), indicating that longitudinal changes were similar between treatments (Table 2). At 6 months, PD did not differ between groups (adjusted mean difference −0.11 mm; *p* = 0.68). However, at 5 years, the particulate group exhibited significantly shallower probing depths than the block group (adjusted mean difference −0.58 mm, 95% CI −1.07 to −0.08; *p* = 0.024), indicating a more favorable long‐term peri‐implant soft‐tissue profile with particulate grafting (Table 2).

Plaque index (PI) remained low and stable in both groups throughout follow‐up. No significant group, time, or interaction effects were observed (overall model *p* = 0.51), with no differences between treatments at either 6 months (adjusted mean difference −0.11; *p* = 0.20) or 5 years (adjusted mean difference ≈0; *p* = 1.00), indicating comparable plaque control across groups (Table 2).

Keratinized mucosa (KM) was significantly greater in the particulate group at 6 months (adjusted mean difference 0.73 mm, 95% CI 0.01–1.45; *p* = 0.048), but this difference was no longer evident at 5 years (adjusted mean difference 0.41 mm; *p* = 0.27). No significant group‐by‐time interaction was detected (*p* = 0.26) (Table 2).

Bleeding on probing (BOP) remained low in both groups and did not differ at 6 months (adjusted mean difference 0.00; *p* = 0.997) or at 5 years (adjusted mean difference −0.02; *p* = 0.84). No significant group, time, or interaction effects were observed (overall model *p* = 0.96), indicating similar peri‐implant inflammatory status between treatments throughout follow‐up (Table 2). Peri‐implant mucositis was diagnosed in 9 of 22 (40.9%) in group Block and in 8 of 21 (38.1%) in group particulate. No cases of peri‐implantitis were identified in either group.

## 4 Discussion

This RCT evaluated the long‐term dimensional stability of buccal hard tissue following GBR of peri‐implant dehiscence defects using either DBBM blocks or particulate DBBM. The previously published 6‐month results demonstrated superior early buccal bone preservation with block grafts (Benic et al. 2019). The present 5‐year findings confirm that these differences were maintained over time.

Overall, the findings indicate that: (1) DBBM blocks preserve significantly more buccal hard‐tissue thickness at both the implant shoulder and the 45° corono‐bucal region, (2) particulate DBBM exhibited pronounced early dimensional reduction with minimal subsequent change and (3) similar peri‐implant clinical parameters.

Although both treatments demonstrated buccal bone reduction over time, the timing and magnitude differed. The particulate group showed near‐complete loss of buccal thickness during early healing, followed by relative stability. In contrast, block grafts resisted early collapse but underwent gradual remodeling during long‐term follow‐up.

These findings are consistent with established biological principles of GBR, which emphasize the importance of mechanical stability and space maintenance during early wound healing. Particulate grafts behave as loosely packed particles that are susceptible to compression under flap tension. In contrast, block grafts provide structural rigidity, maintaining contour and resisting deformation during early healing (Wang and Boyapati 2006). Preclinical and in vitro studies have shown that particulates are vulnerable to compression during flap closure, whereas block‐shaped grafts better withstand soft‐tissue forces (Mir‐Mari et al. 2017, 2016; Schwarz et al. 2008). Clinically, adequate graft stabilization may enhance clot stability and subsequent osteogenesis (Hammerle et al. 1995; An et al. 2022). The present trial extends these findings by demonstrating that early mechanical advantages of block grafts are associated with sustained long‐term dimensional benefits.

**FIGURE A1:**
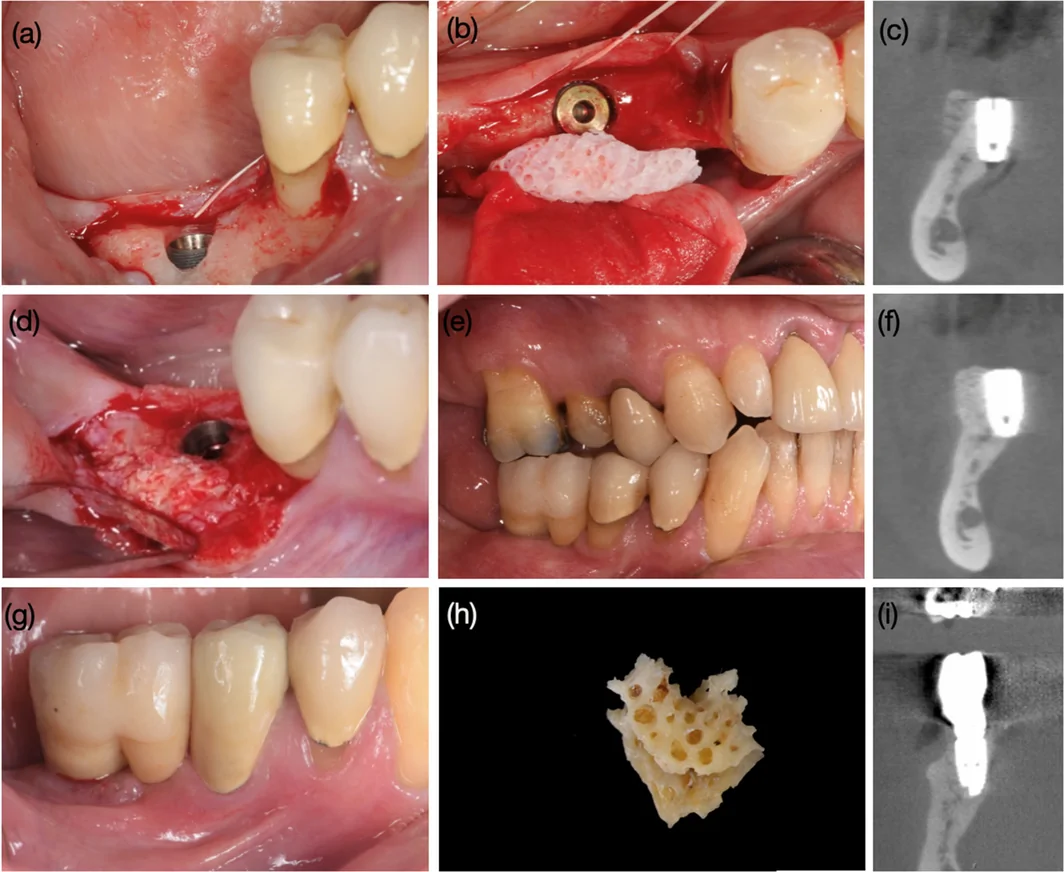
(a) Buccal bone dehiscence after implant placement. Guided bone regeneration with (b) an individually shaped block of deproteinized bovine‐derived bone mineral prior to coverage with a resorbable collagen membrane stabilized with polylactide pins. (c) CBCT bucco‐oral image immediately after wound closure, (f) after 6 months and after (i) 5 years. (d) Complete vertical defect resolution at re‐entry surgery, 6 months after implant placement. Clinical buccal site of the final situation after (e) 1 year and (g) after 5 years. (h) late partial graft separation, resembling sequestra, which was removed.

**FIGURE A2:**
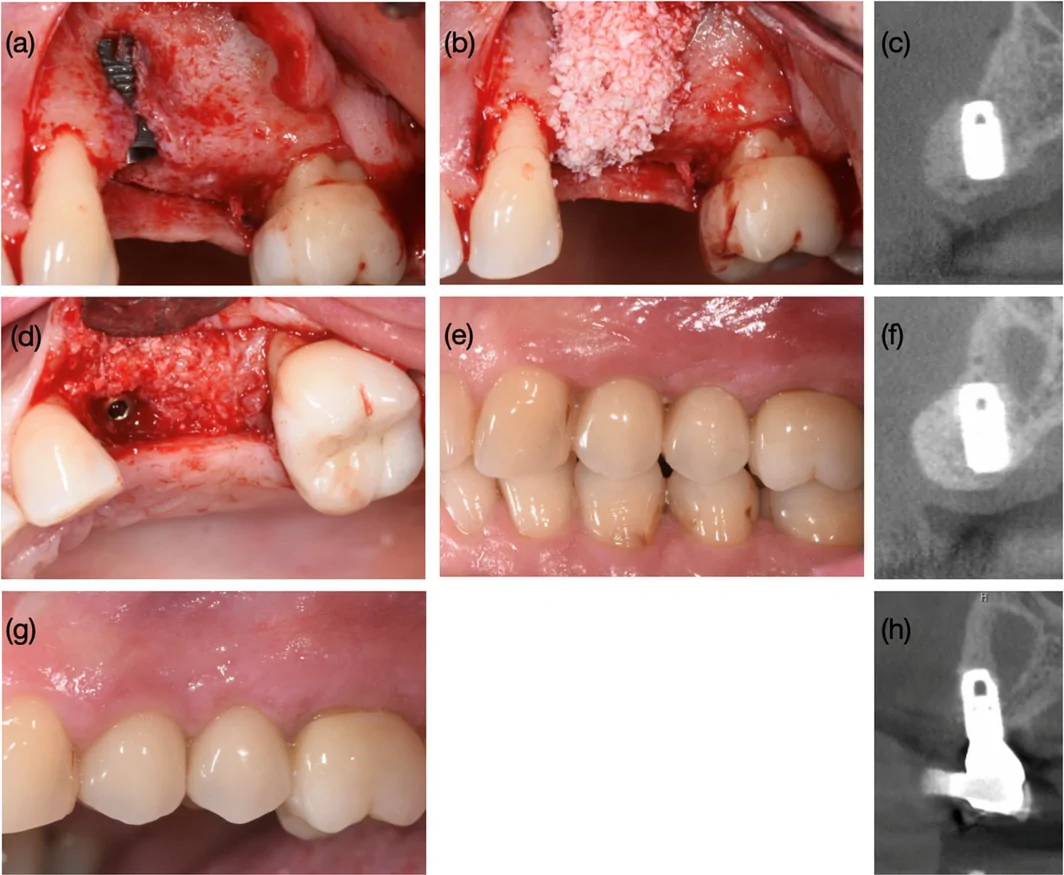
(a) Buccal bone dehiscence after implant placement. Guided bone regeneration with (b) particulate deproteinized bovine‐derived bone mineral, prior to coverage with a resorbable collagen membrane stabilized with polylactide pins. (c) CBCT bucco‐oral image immediately after wound closure, (f) after 6 months and after (i) 5 years. (d) Complete vertical defect resolution at re‐entry surgery, 6 months after implant placement. Clinical buccal site of the final situation after (e) 1 year and (g) after 5 years. (h) Loss of the augmented particulate deproteinized bovine‐derived bone mineral at implant site.

Notably, two cases in the block group showed late graft separation (Figure A1), resembling sequestra, which were removed without compromising implant stability or function. This finding likely reflects the slow remodeling behavior of DBBM blocks, which are known to integrate slowly and may persist as non‐vital scaffold material for extended periods. Previous histologic analyses from this cohort (Benic et al. 2017) showed limited early graft remodeling of DBBM blocks, which may explain this late detachment of residual fragments without affecting implant stability. Similar late block sequestration has been reported, particularly in sites with limited vascular supply or incomplete integration (Schwarz et al. 2010). Clinically, in both cases of late graft separation, no perceptible change in the mid‐facial mucosal contour was noted by the treating clinicians during follow‐up and the patients did not report any subjective change in esthetic appearance; both implant‐supported crowns remained in stable function.

In contrast, the pronounced early reduction in buccal bone thickness observed in the particulate group likely reflects a combination of mechanical compression and biological remodeling processes, including granule displacement and gradual substitution (Araujo et al. 2005). These findings are in line with clinical studies showing improved volume stability when GBR is performed under conditions of enhanced mechanical resistance. For example, titanium‐reinforced non‐resorbable membrane have demonstrated more stable bone augmentations than collagen membranes (Naenni et al. 2020; Strauss et al. 2025). Conversely, a RCT using mechanically weaker polyethylene glycol membranes, have been associated with early graft collapse (Jung et al. 2020). Although these studies modified membrane characteristics rather than the graft material, they support the same biological principle: mechanical stability during early healing is a decisive determinant for long‐term augmentation outcomes. In the present study, the only variable was biomaterial used, with DBBM blocks providing inherent structural stability not achieved by particulate DBBM.

Defect morphology and implant placement timing also merits consideration. Most implants were placed in healed sites (type 3 or 4) resulting predominantly in non‐contained, one‐wall peri‐implant dehiscence defects. Such defects rely heavily on mechanical space maintenance, particularly in the posterior region where thicker mucosa and stronger muscular forces may increase the risk of graft compression after wound closure. Previous clinical studies have shown that non‐contained defects benefit from block‐shaped grafts or additional mechanical reinforcement compared with contained defects (Benic et al. 2016; Lee et al. 2019). These observations are in line with established concepts indicating that defect configuration, specifically the presence of residual bony walls, is more critical for regenerative outcomes than defect size alone (Benic and Hammerle 2014; Fu and Wang 2011; Wang and Boyapati 2006). Defects with more remaining walls create a naturally protected compartment that supports bone formation and stabilizes graft particles over time. In contrast, in non‐contained defects, long‐term contour preservation depends largely on the mechanical properties of the graft and membrane (Song et al. 2023), which can explain the pronounced differences observed in the present trial. Another possible explanation for the observed differences is individual bone phenotype. It has been proposed that augmentation exceeding a patient's phenotypic boundary may be prone to resorption, with the ridge returning towards its original, phenotype‐determined dimension (Quirynen et al. 2023).

Clinical parameters were favorable in both groups, and no peri‐implantitis or implant loss occurred over the 5 years. The absence of peri‐implantitis in the particulate group, despite near‐complete loss of the buccal bone plate, is consistent with clinical evidence showing that posterior implants can remain stable even without buccal bone coverage (Benic et al. 2012; Monje et al. 2023; Schropp et al. 2015) (Derks and Tomasi 2015; Jung et al. 2017; Waller et al. 2020; Zuercher et al. 2023). In such situations soft tissue often adapts and thickens, forming a stable peri‐implant mucosa seal. Some studies have suggested that soft‐tissue thickness, particularly ≥ 2 mm, may be more critical than buccal bone presence, for maintaining peri‐implant health and marginal bone stability (Linkevicius et al. 2015; Waller et al. 2020; Zuercher et al. 2023). In posterior sites with esthetic demands and naturally thicker mucosa, this soft‐tissue compensation may be sufficient to maintain clinical health even in the absence of buccal bone (Zuercher et al. 2023). It is conceivable that the peri‐implant soft tissue may have acted as a compensatory barrier in these sites, in particular in posterior locations where the mucosa is naturally thicker. However, direct radiographic quantification of mucosal thickness was not feasible in the present CBCT dataset, as no soft‐tissue retraction lip was applied during scanning, which is a recognized requirement for reliably differentiating the buccal mucosa from the surrounding soft tissues (Benic et al. 2015). The initial gingival biotype was not systematically recorded at baseline in the present trial. These two aspects represent additional limitations of the present analysis.

The present study has several limitations. First, the sample size was relatively small and the inherent CBCT artefacts may have under‐ or overestimated the presence of bone (Sancho‐Puchades et al. 2015; Vanderstuyft et al. 2019). Second, a quantitative assessment of peri‐implant mucosal thickness from the CBCT data was not feasible, as no soft‐tissue retraction lip was applied during scanning (Benic et al. 2015) and the initial gingival biotype was not systematically recorded. Finally, no objective aesthetic evaluations using the Pink Esthetic Score were performed.

## 5 Conclusions

GBR using either DBBM blocks or particulate DBBM resulted in comparable clinical outcomes including marginal bone levels, peri‐implant health and implant survival. However, DBBM blocks provided superior long‐term preservation of the buccal hard‐tissue contour. Future studies should further assess surgical time, costs, patient‐reported outcomes and longer‐term follow‐up to determine the optimal approach from clinical, economic and patient‐centered perspectives.

## Author Contributions

**Erkan Evci:** software, investigation, validation, formal analysis. **Goran Benic:** conceptualization, methodology, supervision, funding acquisition, project administration, writing – review and editing. **Anina N. Zuercher:** software, investigation, writing – review and editing, validation, formal analysis, writing – original draft, visualization. **Riccardo D. Kraus:** software, investigation, validation, formal analysis. **Ronald E. Jung:** conceptualization, methodology, supervision, funding acquisition, resources. **Franz J. Strauss:** software, investigation, writing – review and editing, validation, formal analysis, visualization, writing – original draft.

## Funding

This work was supported by Osteology Foundation, 10‐026.

## Conflicts of Interest

The authors declare no conflicts of interest.

## Supporting information


**Data S1:** CONSORT 2025 checklist of information to include when reporting a randomized trial.

**Table S1:** Patient demographics, implant site distribution and baseline defect dimensions per treatment group.

## Data Availability

The data that support the findings of this study are available from the corresponding author upon reasonable request.

## References

[clr70150-bib-0038] An, Y. Z. , F. J. Strauss , J. Y. Park , Y. Q. Shen , D. S. Thoma , and J. S. Lee . 2022. “Membrane Fixation Enhances Guided Bone Regeneration in Standardized Calvarial Defects: A Pre‐Clinical Study.” Journal of Clinical Periodontology 49, no. 2: 177–187. 10.1111/jcpe.13583. 34866208

[clr70150-bib-0001] Araujo, M. G. , F. Sukekava , J. L. Wennstrom , and J. Lindhe . 2005. “Ridge Alterations Following Implant Placement in Fresh Extraction Sockets: An Experimental Study in the Dog.” Journal of Clinical Periodontology 32, no. 6: 645–652. 10.1111/j.1600-051X.2005.00726.x. 15882225

[clr70150-bib-0002] Benic, G. I. , B. M. Eisner , R. E. Jung , T. Basler , D. Schneider , and C. H. F. Hammerle . 2019. “Hard Tissue Changes After Guided Bone Regeneration of Peri‐Implant Defects Comparing Block Versus Particulate Bone Substitutes: 6‐Month Results of a Randomized Controlled Clinical Trial.” Clinical Oral Implants Research 30, no. 10: 1016–1026. 10.1111/clr.13515. 31332835

[clr70150-bib-0003] Benic, G. I. , M. Elmasry , and C. H. Hämmerle . 2015. “Novel Digital Imaging Techniques to Assess the Outcome in Oral Rehabilitation With Dental Implants: A Narrative Review.” Clinical Oral Implants Research 26, no. Suppl 11: 86–96. 10.1111/clr.12616. 26010421

[clr70150-bib-0004] Benic, G. I. , and C. H. Hammerle . 2014. “Horizontal Bone Augmentation by Means of Guided Bone Regeneration.” Periodontology 2000 66, no. 1: 13–40. 10.1111/prd.12039. 25123759

[clr70150-bib-0005] Benic, G. I. , M. Mokti , C. J. Chen , H. P. Weber , C. H. Hammerle , and G. O. Gallucci . 2012. “Dimensions of Buccal Bone and Mucosa at Immediately Placed Implants After 7 Years: A Clinical and Cone Beam Computed Tomography Study.” Clinical Oral Implants Research 23, no. 5: 560–566. 10.1111/j.1600-0501.2011.02253.x. 22093013

[clr70150-bib-0006] Benic, G. I. , D. S. Thoma , R. E. Jung , et al. 2017. “Guided Bone Regeneration With Particulate vs. Block Xenogenic Bone Substitutes: A Pilot Cone Beam Computed Tomographic Investigation.” Clinical Oral Implants Research 28, no. 11: e262–e270. 10.1111/clr.13011. 28378530

[clr70150-bib-0007] Benic, G. I. , D. S. Thoma , F. Munoz , I. Sanz Martin , R. E. Jung , and C. H. Hammerle . 2016. “Guided Bone Regeneration of Peri‐Implant Defects With Particulated and Block Xenogenic Bone Substitutes.” Clinical Oral Implants Research 27, no. 5: 567–576. 10.1111/clr.12625. 26073212

[clr70150-bib-0008] Berglundh, T. , G. Armitage , M. G. Araujo , et al. 2018. “Peri‐Implant Diseases and Conditions: Consensus Report of Workgroup 4 of the 2017 World Workshop on the Classification of Periodontal and Peri‐Implant Diseases and Conditions.” Journal of Clinical Periodontology 45, no. Suppl 20: S286–s291. 10.1111/jcpe.12957. 29926491

[clr70150-bib-0009] Dahlin, C. , A. Linde , J. Gottlow , and S. Nyman . 1988. “Healing of Bone Defects by Guided Tissue Regeneration.” Plastic and Reconstructive Surgery 81, no. 5: 672–676. 10.1097/00006534-198805000-00004. 3362985

[clr70150-bib-0010] De Bruyckere, T. , J. Cosyn , F. Younes , et al. 2020. “A Randomized Controlled Study Comparing Guided Bone Regeneration With Connective Tissue Graft to Re‐Establish Buccal Convexity: One‐Year Aesthetic and Patient‐Reported Outcomes.” Clinical Oral Implants Research 31, no. 6: 507–516. 10.1111/clr.13587. 32011032

[clr70150-bib-0011] Derks, J. , and C. Tomasi . 2015. “Peri‐Implant Health and Disease. A Systematic Review of Current Epidemiology.” Journal of Clinical Periodontology 42 Suppl 16: S158–S171. 10.1111/jcpe.12334. 25495683

[clr70150-bib-0012] Fu, J. H. , and H. L. Wang . 2011. “Horizontal Bone Augmentation: The Decision Tree.” International Journal of Periodontics & Restorative Dentistry 31, no. 4: 429–436. 21837309

[clr70150-bib-0013] Hammerle, C. H. , J. Schmid , N. P. Lang , and A. J. Olah . 1995. “Temporal Dynamics of Healing in Rabbit Cranial Defects Using Guided Bone Regeneration.” Journal of Oral and Maxillofacial Surgery 53, no. 2: 167–174. 10.1016/0278-2391(95)90396-8. 7830183

[clr70150-bib-0014] Herrera, D. , T. Berglundh , F. Schwarz , et al. 2023. “Prevention and Treatment of Peri‐Implant Diseases‐The EFP S3 Level Clinical Practice Guideline.” Journal of Clinical Periodontology 50, no. Suppl 26: 4–76. 10.1111/jcpe.13823. 37271498

[clr70150-bib-0015] Jung, R. E. , M. Herzog , K. Wolleb , C. F. Ramel , D. S. Thoma , and C. H. Hammerle . 2017. “A Randomized Controlled Clinical Trial Comparing Small Buccal Dehiscence Defects Around Dental Implants Treated With Guided Bone Regeneration or Left for Spontaneous Healing.” Clinical Oral Implants Research 28, no. 3: 348–354. 10.1111/clr.12806. 26923088

[clr70150-bib-0016] Jung, R. E. , I. Mihatovic , L. Cordaro , et al. 2020. “Comparison of a Polyethylene Glycol Membrane and a Collagen Membrane for the Treatment of Bone Dehiscence Defects at Bone Level Implants‐A Prospective, Randomized, Controlled, Multicenter Clinical Trial.” Clinical Oral Implants Research 31, no. 11: 1105–1115. 10.1111/clr.13657. 32875638

[clr70150-bib-0017] Lee, J. , D. Park , K. T. Koo , Y. J. Seol , and Y. M. Lee . 2019. “Validity of a Regenerative Procedure for a Minor Bone Defect With Immediate Implant Placement: A Systematic Review and Meta‐Analysis.” Acta Odontologica Scandinavica 77, no. 2: 99–106. 10.1080/00016357.2018.1508743. 30600736

[clr70150-bib-0018] Linkevicius, T. , A. Puisys , L. Linkeviciene , V. Peciuliene , and M. Schlee . 2015. “Crestal Bone Stability Around Implants With Horizontally Matching Connection After Soft Tissue Thickening: A Prospective Clinical Trial.” Clinical Implant Dentistry and Related Research 17, no. 3: 497–508. 10.1111/cid.12155. 24103157

[clr70150-bib-0019] Mellonig, J. T. , M. Nevins , and R. Sanchez . 1998. “Evaluation of a Bioabsorbable Physical Barrier for Guided Bone Regeneration. Part II. Material and a Bone Replacement Graft.” International Journal of Periodontics & Restorative Dentistry 18, no. 2: 129–137. 9663092

[clr70150-bib-0020] Mir‐Mari, J. , G. I. Benic , E. Valmaseda‐Castellon , C. H. F. Hammerle , and R. E. Jung . 2017. “Influence of Wound Closure on the Volume Stability of Particulate and Non‐Particulate GBR Materials: An in Vitro Cone‐Beam Computed Tomographic Examination. Part II.” Clinical Oral Implants Research 28, no. 6: 631–639. 10.1111/clr.12845. 27060694

[clr70150-bib-0021] Mir‐Mari, J. , H. Wui , R. E. Jung , C. H. Hammerle , and G. I. Benic . 2016. “Influence of Blinded Wound Closure on the Volume Stability of Different GBR Materials: An in Vitro Cone‐Beam Computed Tomographic Examination.” Clinical Oral Implants Research 27, no. 2: 258–265. 10.1111/clr.12590. 25856209

[clr70150-bib-0022] Monje, A. , A. Roccuzzo , D. Buser , and H. L. Wang . 2023. “Influence of Buccal Bone Wall Thickness on the Peri‐Implant Hard and Soft Tissue Dimensional Changes: A Systematic Review.” Clinical Oral Implants Research 34, no. 3: 157–176. 10.1111/clr.14029. 36626118

[clr70150-bib-0023] Naenni, N. , H. C. Lim , F. J. Strauss , R. E. Jung , C. H. F. Hammerle , and D. S. Thoma . 2020. “Local Tissue Effects of Various Barrier Membranes in a Rat Subcutaneous Model.” Journal of Periodontal & Implant Science 50, no. 5: 327–339. 10.5051/jpis.2000380019. 33124210 PMC7606894

[clr70150-bib-0024] Omar, O. , I. Elgali , C. Dahlin , and P. Thomsen . 2019. “Barrier Membranes: More Than the Barrier Effect?” Journal of Clinical Periodontology 46, no. Suppl 21: 103–123. 10.1111/jcpe.13068. 30667525 PMC6704362

[clr70150-bib-0025] Quirynen, M. , P. Lahoud , W. Teughels , et al. 2023. “Individual “Alveolar Phenotype” Limits Dimensions of Lateral Bone Augmentation.” Journal of Clinical Periodontology 50, no. 4: 500–510. 10.1111/jcpe.13764. 36574768

[clr70150-bib-0026] Sancho‐Puchades, M. , C. H. Hammerle , and G. I. Benic . 2015. “In Vitro Assessment of Artifacts Induced by Titanium, Titanium‐Zirconium and Zirconium Dioxide Implants in Cone‐Beam Computed Tomography.” Clinical Oral Implants Research 26, no. 10: 1222–1228. 10.1111/clr.12438. 25040484

[clr70150-bib-0027] Schropp, L. , A. Wenzel , R. Spin‐Neto , and A. Stavropoulos . 2015. “Fate of the Buccal Bone at Implants Placed Early, Delayed, or Late After Tooth Extraction Analyzed by Cone Beam CT: 10‐Year Results From a Randomized, Controlled, Clinical Study.” Clinical Oral Implants Research 26, no. 5: 492–500. 10.1111/clr.12424. 24890861

[clr70150-bib-0028] Schwarz, F. , R. E. Jung , T. Fienitz , M. Wieland , J. Becker , and M. Sager . 2010. “Impact of Guided Bone Regeneration and Defect Dimension on Wound Healing at Chemically Modified Hydrophilic Titanium Implant Surfaces: An Experimental Study in Dogs.” Journal of Clinical Periodontology 37, no. 5: 474–485. 10.1111/j.1600-051X.2010.01551.x. 20507370

[clr70150-bib-0029] Schwarz, F. , D. Rothamel , M. Herten , et al. 2008. “Immunohistochemical Characterization of Guided Bone Regeneration at a Dehiscence‐Type Defect Using Different Barrier Membranes: An Experimental Study in Dogs.” Clinical Oral Implants Research 19, no. 4: 402–415. 10.1111/j.1600-0501.2007.01486.x. 18324961

[clr70150-bib-0030] Song, Y. W. , S. P. Bienz , G. I. Benic , et al. 2023. “Soft‐Tissue Dimensional Change Following Guided Bone Regeneration on Peri‐Implant Defects Using Soft‐Type Block or Particulate Bone Substitutes: 1‐Year Outcomes of a Randomized Controlled Clinical Trial.” Journal of Clinical Periodontology 50, no. 2: 147–157. 10.1111/jcpe.13738. 36330670

[clr70150-bib-0031] Strauss, F. J. , D. Schneider , R. E. Jung , R. Kraus , D. S. Thoma , and N. Naenni . 2025. “Hard and Soft Tissue Contour Changes Following Simultaneous Guided Bone Regeneration at Single Peri‐Implant Dehiscence Defects Using Either Resorbable or Non‐Resorbable Membranes: A 6‐Month Secondary Analysis of a Randomized Controlled Trial.” Clinical Oral Investigations 29, no. 5: 231. 10.1007/s00784-025-06322-4. 40199760 PMC11978683

[clr70150-bib-0032] Thoma, D. S. , S. P. Bienz , E. Figuero , R. E. Jung , and I. Sanz‐Martin . 2019. “Efficacy of Lateral Bone Augmentation Performed Simultaneously With Dental Implant Placement: A Systematic Review and Meta‐Analysis.” Journal of Clinical Periodontology 46, no. Suppl 21: 257–276. 10.1111/jcpe.13050. 30675733

[clr70150-bib-0033] Tonetti, M. S. , M. Sanz , G. Avila‐Ortiz , et al. 2023. “Relevant Domains, Core Outcome Sets and Measurements for Implant Dentistry Clinical Trials: The Implant Dentistry Core Outcome Set and Measurement (ID‐COSM) International Consensus Report.” Journal of Clinical Periodontology 50, no. Suppl 25: 5–21. 10.1111/jcpe.13808. 37143289

[clr70150-bib-0034] Vanderstuyft, T. , M. Tarce , B. Sanaan , R. Jacobs , K. de Faria Vasconcelos , and M. Quirynen . 2019. “Inaccuracy of Buccal Bone Thickness Estimation on Cone‐Beam CT due to Implant Blooming: An Ex‐Vivo Study.” Journal of Clinical Periodontology 46, no. 11: 1134–1143. 10.1111/jcpe.13183. 31446644

[clr70150-bib-0035] Waller, T. , M. Herzog , D. S. Thoma , J. Husler , C. H. F. Hammerle , and R. E. Jung . 2020. “Long‐Term Clinical and Radiographic Results After Treatment or no Treatment of Small Buccal Bone Dehiscences at Posterior Dental Implants: A Randomized, Controlled Clinical Trial.” Clinical Oral Implants Research 31, no. 6: 517–525. 10.1111/clr.13588. 32011015

[clr70150-bib-0036] Wang, H. L. , and L. Boyapati . 2006. “‘PASS’ Principles for Predictable Bone Regeneration.” Implant Dentistry 15, no. 1: 8–17. 10.1097/01.id.0000204762.39826.0f. 16569956

[clr70150-bib-0037] Zuercher, A. N. , F. J. Strauss , P. N. Paque , S. P. Bienz , R. E. Jung , and D. S. Thoma . 2023. “Randomized Controlled Pilot Study Comparing Small Buccal Defects Around Dental Implants Treated With a Subepithelial Connective Tissue Graft or With Guided Bone Regeneration.” Clinical Oral Implants Research 34, no. 10: 1094–1105. 10.1111/clr.14140. 37483129

