# Supplementary material for: Block Versus Particulate Deproteinized Bovine Bone Mineral for Guided Bone Regeneration of Peri‐Implant Dehiscence Defects: A 5‐Year Randomized Controlled Trial
Source: Clin Oral Implants Res. 2026 Jun 28;37(9):1096–108. doi: 10.1111/clr.70150 (PMC13542762; doi:10.1111/clr.70150)
Supplement: Supplementary file 2 [file CLR-37-1096-s002.docx]

**Supplementary Table S1 -** Patient demographics, implant site distribution and baseline defect dimensions per treatment group.

|  | | **Group Block** | | **Group Particulate** | |
| --- | --- | --- | --- | --- | --- |
| **Parameter** | **Category** | **Mean (SD) / n (%)** | **Median (Q1; Q3)** | **Mean (SD) / n (%)** | **Median (Q1; Q3)** |
| **Sex** | Female | 7 (58.3%) | — | 7 (58.3%) | — |
|  | Male | 5 (41.7%) | — | 5 (41.7%) | — |
| **Age (years)** | Baseline | 62.0 (10.0) | 62.8 (43.5; 78.6) | 58.1 (14.5) | 62.2 (28.7; 78.8) |
| **Jaw** | Maxilla | 7 (58.3%) | — | 6 (50.0%) | — |
|  | Mandible | 5 (41.7%) | — | 6 (50.0%) | — |
| **Tooth position** | Incisive | 4 (33.3%) | — | 0 (0.0%) | — |
|  | Canine | 0 (0.0%) | — | 3 (25.0%) | — |
|  | Premolar | 5 (41.7%) | — | 9 (75.0%) | — |
|  | Molar | 3 (25.0%) | — | 0 (0.0%) | — |
| **Vertical dehiscence height (mm)** | Baseline | 4.5 (2.5) | 3.5 (2.0; 9.0) | 4.6 (2.1) | 4.3 (2.0; 8.0) |

*Note: Continuous variables are reported as mean (SD) and median (Q1; Q3) and were compared by Welch t-test on raw values; categorical variables are reported as n (%) and were compared by χ²-test. p-values are reported.*

Vertical dehiscence height was measured intra-operatively at the time of implant placement as the distance from the implant shoulder to the most coronal level of buccal bone, in accordance with the parent trial (Benic et al., 2019). Horizontal defect width was not systematically recorded in the parent study database. All implants were of the same type (AstraTech / OsseoSpeed EV, Dentsply Sirona Implants); individual implant diameter and length were chosen by the treating surgeon according to the available bone volume and prosthetic needs and were not standardized. Demographic and site distribution data are consistent with those reported in the parent trial (Benic et al., 2019, Table A1).

*Abbreviations: SD, standard deviation; Q1, first quartile; Q3, third quartile; CI, confidence interval; n/a, not applicable.*
